# Specific associations between heart rate variability and motor domains in children with attention-deficit/hyperactivity disorder: a comparative study

**DOI:** 10.3389/fpsyt.2026.1786613

**Published:** 2026-04-28

**Authors:** Jiaxin Chen, Xiaoxiao Chen, Deqiang Zhao, Chunmiao Wang, Haixia Hu, Yanfeng Zhang

**Affiliations:** 1 China Institute of Sport Science, National Physical Fitness and Fitness-for-All Research Center, Beijing, China; 2 Jinzhou Medical University, College of Physical Education and Sports Rehabilitation, Jinzhou, Liaoning, China; 3 Ai You Dong Institute of Child and Adolescent Sports and Health, Weifang, Shandong, China

**Keywords:** attention-deficit/hyperactivity disorder, autonomic nervous system, heart rate variability, motor proficiency, SDNN

## Abstract

**Objective:**

This study compared children with Attention-Deficit/Hyperactivity Disorder (ADHD) and typically developing (TD) controls on heart rate variability (HRV) and motor proficiency, and examined their combined role in predicting ADHD symptom severity.

**Methods:**

Twenty-nine children with ADHD and 33 TD children, matched for age and sex, underwent assessment. ADHD symptoms were assessed using the Strengths and Weaknesses of ADHD Symptoms and Normal Behavior (SWAN) scale, motor skills were evaluated using the Movement Assessment Battery for Children-2 (MABC-2), and resting-state HRV was recorded via a 5-minute electrocardiogram.

**Results:**

The ADHD group demonstrated significantly poorer performance across all MABC-2 domains (fine motor, coordination, balance, total score; all *P* < 0.05). They also exhibited altered autonomic nervous system function, evidenced by significantly lower log-transformed Root Mean Square of Successive Differences and Standard Deviation of Normal-to-Normal Intervals, and a higher log-transformed LF/HF ratio (all *P* < 0.05). Correlation analyses revealed significant associations between motor coordination and HRV indices (logSDNN, logRMSSD). Hierarchical regression analysis identified logSDNN as a significant independent predictor of ADHD symptom severity, even after controlling for age, sex, and motor abilities.

**Conclusion:**

Children with ADHD show concurrent deficits in motor skills and autonomic regulation. HRV, particularly SDNN, serves as a robust physiological marker for symptom severity, suggesting that integrated assessment of motor and autonomic function provides a more comprehensive understanding of ADHD.

## 1 Introduction

Attention-Deficit/Hyperactivity Disorder (ADHD) is one of the most common neurodevelopmental disorders in childhood, affecting approximately 5% to 10% of children globally (1, 2). Its core clinical features include a developmentally disproportionate pattern of inattention, hyperactivity, and/or impulsivity (3, 4), significantly impacting children’s academic performance, social interactions, and daily functioning (5–7). Traditionally viewed primarily as a behavioral and cognitive disorder (8), growing evidence from neuroscience indicates that the pathophysiology of ADHD involves broader neurophysiological systems, particularly dysfunction of the Autonomic Nervous System (ANS) (9).

Heart Rate Variability (HRV), a non-invasive physiological indicator quantifying autonomic nervous function, has been increasingly used in research on psychiatric and neurodevelopmental disorders (10–12). HRV reflects the balance between sympathetic and parasympathetic nervous activity and the functional integrity of the heart-brain axis (13, 14). It is considered a vital physiological substrate for emotion regulation, executive function, and stress adaptability. Among commonly used time-domain HRV indices, SDNN and RMSSD are particularly informative. SDNN (standard deviation of normal-to-normal intervals) reflects the overall variability of heart rate across the recording period and is generally considered an index of global autonomic flexibility. RMSSD (root mean square of successive differences) primarily reflects short-term beat-to-beat variability and is widely regarded as a marker of parasympathetic, or vagally mediated, cardiac regulation. Lower values of SDNN or RMSSD are typically interpreted as indicating reduced autonomic adaptability and weaker self-regulatory capacity. Because self-regulation deficits are central to ADHD, these indices are especially relevant for understanding the physiological basis of symptom expression. Numerous studies have reported abnormal HRV indices in children/adolescents with ADHD, such as reduced Root Mean Square of Successive Differences (RMSSD) and Standard Deviation of Normal-to-Normal Intervals (SDNN) or altered LF/HF ratio (15), suggesting impaired autonomic regulation, notably reduced parasympathetic activity. However, existing findings are inconsistent (16). Furthermore, research has predominantly focused on the associations between HRV and cognitive/emotional outcomes, with relatively few studies investigating the link between HRV and motor development.

Motor development, encompassing domains like fine motor skills, coordination, and balance control, is a crucial behavioral phenotype reflecting motor control and neural maturation (17). Its maturation relies on the synergistic functioning of neural circuits involving the prefrontal cortex, cerebellum, and basal ganglia. Motor coordination difficulties are highly prevalent in children with ADHD; approximately half exhibit clinically significant motor impairments (18, 19). This suggests that motor development issues may be a concomitant feature of ADHD rather than merely a consequence of inattention or hyperactivity. Few existing studies have incorporated both motor proficiency and HRV into predictive models. Should a stable association between HRV and motor development exist, it would suggest that autonomic regulation may influence behavioral output by modulating central motor control networks. This perspective offers a novel framework for understanding the heterogeneity of ADHD and identifying potential biomarkers.

The theoretical basis for the interaction between autonomic regulation and motor control lies in the Neurovisceral Integration Model. Central to this model is the Central Autonomic Network (CAN), which includes the prefrontal cortex (PFC), anterior cingulate cortex (ACC), and amygdala. These brain regions not only regulate autonomic outflow via the vagus nerve but also play a critical role in high-level motor planning and inhibitory control. Specifically, the ACC serves as a functional hub that integrates physiological arousal with motor execution. In ADHD, structural or functional deficits within these shared neural circuits may lead to a ‘top-down’ dysregulation, where poor autonomic ‘braking’ (indexed by low HRV) coincides with failed motor inhibition and coordination (20).

We define the ‘motor-autonomic-behavioral’ perspective as an integrated framework wherein physiological arousal (autonomic) and physical coordination (motor) are viewed as intertwined regulatory processes that jointly manifest as the behavioral symptoms of ADHD. From this perspective, motor and autonomic deficits are not isolated comorbidities but are phenotypic expressions of a common underlying neurobiological impairment in self-regulation (21).

The discovery of these associations would support the hypothesis that ADHD is a multi-system regulatory disorder rather than a purely cognitive or behavioral one. It would provide evidence that the underlying pathophysiology involves a generalized deficit in the integration of internal physiological states with external motoric demands, likely localized to dysfunctions in the prefrontal-subcortical pathways. This suggests that autonomic biomarkers could potentially serve as objective indicators of the integrity of motor and behavioral control networks in children with ADHD.

Based on this background, this study aims to systematically explore the specific associations between HRV and various domains of motor development in children with ADHD and, through comparison with typically developing children, reveal their independent and combined roles in ADHD symptom expression. We hypothesize that (1): The ADHD group will exhibit decreased parasympathetic activity and sympathetic dominance on HRV indices compared to the TD group (2); HRV indices will be significantly correlated with fine motor skills, coordination, and balance as assessed by the MABC-2; (3) After controlling for the effects of age and sex, HRV will provide significant additional explanatory power for ADHD symptom severity. This study expects to provide new empirical evidence for the pathophysiology and clinical assessment of ADHD from a multi-dimensional “motor-autonomic-behavioral” integrative perspective.

## 2 Methods

### 2.1 Participants

*A priori* sample size estimation was performed using G*Power software (22), based on an independent samples t-test framework. Parameters were set as follows: significance level (α) = 0.05 (two-tailed), statistical power (1-β) = 0.80, and effect size (Cohen’s d) = 0.7 (moderate to large). The calculation indicated that a minimum of 26 participants per group (total N = 52) was required to detect this effect size. This study ultimately recruited 33 children for the ADHD group (final valid N = 29, males=26, females=3) and 33 children for the typically developing (TD) control group (males=28, females=5). Control children were individually matched to ADHD participants based on age (± 6 months) in a 1:1 ratio, while also striving for comparable sex distribution. Statistical analysis confirmed no significant differences in age or sex between the groups. ADHD diagnosis was based on DSM-5 criteria and a structured clinical interview (23, 24), conducted by experienced child neurologists from Beijing Children’s Hospital, Capital Medical University. Inclusion criteria: age 6–10 years. Exclusion criteria included: ongoing pharmacological treatment; IQ below 70 as measured by the Wechsler Intelligence Scale for Children (25, 26); severe somatic diseases; and hearing/visual impairments. Participants were excluded if they had a history of other neurodevelopmental disorders, such as Autism Spectrum Disorder (ASD) or Learning Disabilities (LD), based on prior clinical diagnoses and parental reports. The study was approved by the Ethics Committee of the China Institute of Sport Science (Approval No.: CISSLA20250320). Written informed consent was obtained from all guardians, and children aged 8 and above additionally provided assent using a child-friendly version.

### 2.2 ADHD symptom assessment

The Strengths and Weaknesses of ADHD Symptoms and Normal Behavior (SWAN) scale was completed by the participants’ parents or legal guardians to evaluate the core symptom severity of ADHD. The scale consists of 18 items based on the diagnostic criteria for ADHD in the Diagnostic and Statistical Manual of Mental Disorders, Fourth Edition (DSM-IV). These items are equally divided into two subscales:

1. Inattention (9 items, which evaluate behaviors such as difficulty sustaining attention, organizing tasks, and following instructions);
2. Hyperactivity/Impulsivity (9 items, which evaluate behaviors such as fidgeting, excessive talking, and difficulty waiting for one’s turn).

Each item is rated on a 7-point Likert scale ranging from -3 (far below average) to +3 (far above average), comparing the child’s behavior with that of typically developing peers of the same age. To facilitate statistical analysis and align with standard clinical scoring, the scores were reverse-coded or summed such that higher scores represent greater symptom severity. In this study, both the subscale scores and the overall total score (the sum of all 18 items) were utilized as continuous measures of ADHD symptom severity. Blume et al. reported good internal reliabilities for the German version (α=0.85–0.90) (27). The English version has demonstrated good convergent and discriminant validity (28).

### 2.3 Motor ability assessment

The Movement Assessment Battery for Children-2 (MABC-2) was used to assess fine motor skills, coordination, and static/dynamic balance (29). Assessments were conducted according to the manual, and scoring was performed by trained evaluators. Raw scores were entered into the system and, considering age and sex factors, were converted into standard scores for the three dimensions: fine motor skills, coordination, and static/dynamic balance. Finally, According to the MABC-2 manual, raw scores for each task were first converted into age-adjusted standard scores (with a mean of 10 and a standard deviation of 3). To facilitate comparison across different measures and accommodate the regression analysis, these standard scores were further linearly transformed into Z-scores (with a mean of 0 and a standard deviation of 1). Higher Z-scores indicate better motor performance. According to the MABC-2 manual, participants were further categorized based on their total test percentiles to provide a clinical interpretation: a percentile ≤5th (corresponding to≤ -1.645) indicates definite motor difficulty (suggestive of DCD), a percentile between 5th and 15th (-1.645 < Z ≤-1.04) indicates an at-risk standing, and a percentile > 15th (Z > -1.04) indicates typical motor development.

### 2.4 Heart rate variability acquisition and analysis

Resting ECG was recorded in a quiet room. Participants sat quietly and rested for 5 minutes initially. HRV data were then recorded for 5 minutes using a Polar H10 chest strap (Polar Oy, Finland) to capture R-R intervals, the accuracy of which has been validated (30). During recording, children followed a paced breathing rhythm (3 seconds inhalation, 3 seconds exhalation) displayed dynamically by Kubios software. Data were recorded using the Kubios HRV application (iOS). Time-domain indices calculated were RMSSD and SDNN. Frequency-domain indices calculated were Low Frequency (LF) and High Frequency (HF) power, with the LF/HF ratio used as an indicator of sympathetic-parasympathetic balance. To reduce skewness, natural logarithmic transformations were applied to RMSSD, SDNN, and LF/HF (resulting in logRMSSD, logSDNN, logLF/HF) for use in statistical analyses (31).

To ensure high-quality data and minimize motion artifacts in children with ADHD, several behavioral support strategies were implemented (1): a 10-minute acclimatization period was provided for children to become familiar with the laboratory environment and the electrode placement; (2) children were instructed to play a “statue game,” which encouraged them to sit as still as possible in a comfortable chair with their hands resting on their laps; (3) parents were permitted to sit quietly beside their children throughout the procedure to reduce situational anxiety and enhance emotional security. The researcher monitored the ECG signal in real-time, and any recordings compromised by excessive movement were restarted after the child reached a stable state.

### 2.5 Statistical methods

All data were analyzed using SPSS 27.0. Initial data cleaning included logical checks, missing value detection, and outlier identification. Continuous variables are described as Mean ± Standard Deviation (Mean ± SD). The Shapiro-Wilk test was used to assess the normality of continuous variables. Due to the skewed distribution of the HRV indices RMSSD, SDNN, and LF/HF, natural logarithms were applied (logRMSSD, logSDNN, logLF/HF) based on common practice in the literature to improve normality for subsequent statistical analyses.

Differences between the ADHD and TD control groups in age, MABC-2 scores, HRV indices, and symptom scores were examined using independent samples t-tests.

To explore the associations between motor development and HRV indices, Pearson correlation analysis was conducted, calculating correlation coefficients (r) and their significance levels. The correlation matrix included symptom scores, fine motor skills, coordination, static/dynamic balance, MABC-2 total score, logRMSSD, logSDNN, and logLF/HF. To control for the inflation of Type I error due to multiple correlations, the False Discovery Rate (FDR) was controlled at 0.05 using the Benjamini-Hochberg procedure.

Hierarchical multiple regression was further employed to determine the unique and incremental contribution of motor domains and HRV to ADHD symptom severity. A three-step model was constructed based on theoretical priority: age and sex were entered in Step 1 as covariates to control for biological maturity; motor domains (Manual Dexterity, Aiming & Catching, and Balance) were entered in Step 2 as behavioral predictors; and HRV parameters (e.g., logSDNN) were entered in Step 3 to evaluate their incremental predictive value beyond demographic and behavioral factors. To ensure model parsimony and avoid multicollinearity, only variables that demonstrated significant correlations with the SWAN total score (after FDR correction) were selected for inclusion in the final model.

All statistical tests were two-tailed, with the significance level set at α = 0.05. Where necessary, 95% confidence intervals and effect size indicators are also reported.

For clarity in interpreting the results: (1) Higher scores on all MABC-2 domains (Manual Dexterity, Aiming & Catching, and Balance) indicate superior motor performance, with negative Z-scores representing performance below the normative mean. (2) For HRV time-domain indices (SDNN and RMSSD), higher values reflect greater parasympathetic activity and better autonomic regulation. (3) In the frequency domain, higher HF power indicates stronger vagal tone, while a higher LF/HF ratio suggests a relative dominance of sympathetic activity or decreased autonomic balance.

## 3 Results

### 3.1 Baseline characteristics and group comparisons

No significant difference was found in age between the ADHD group (n=29) and the TD control group (n=33) (t = -0.18, *P* = 0.86). Regarding symptom levels, the ADHD group scored significantly lower than the control group (t = 7.08, *P* < 0.01), indicating markedly more severe clinical symptoms.

In terms of motor development, the ADHD group scored significantly lower than the control group on fine motor skills, coordination, static/dynamic balance, and the MABC-2 total score (all *P* < 0.05). The differences were most pronounced for fine motor skills (t = 2.88, P < 0.01) and the MABC-2 total score (t = 3.34, *P* < 0.01).Based on these clinical cut-offs, in the ADHD group, 10.3% of the children met the criteria for definite motor difficulty, and 17.3% were categorized as ‘at-risk’.

Regarding heart indicators, resting heart rate showed no significant difference between the groups (*P* = 0.61). However, HRV indices revealed significant group differences: the ADHD group had significantly lower logRMSSD and logSDNN than the control group (t = 2.34, *P* < 0.05; t = 3.26, *P* < 0.01, respectively), and significantly higher logLF/HF (t = -2.14, *P* < 0.05).

These results suggest significantly impaired autonomic nervous system regulatory capacity in children with ADHD. All group comparisons are shown in **Table 1**.

**Table 1:** Group differences in ADHD symptoms, motor development, and heart rate variability between children with ADHD and typically developing controls.

Indicator	TD group (n=33)	ADHD group (n=29)	t-value	*P*-value	95% CI
Age	7.82 ± 0.98	7.86 ± 0.95	-0.18	0.86	-0.54 ~ 0.45
Symptom Score	15.36 ± 5.78	-16.24 ± 2.35	7.08	< 0.01	11.54 ~ 20.94
Fine Motor Skills	0.83 ± 1.95	-1.06 ± 3.13	2.88	< 0.01	0.58 ~ 3.20
Coordination	0.50 ± 1.67	-0.68 ± 1.53	2.88	< 0.01	0.36 ~ 2.00
Static/Dynamic Balance	0.79 ± 2.16	-0.90 ± 3.51	2.25	< 0.05	0.17 ~ 3.20
MABC-2 Total Score	2.12 ± 4.22	-2.41 ± 6.14	3.34	< 0.01	1.79 ~ 7.25
Resting Heart Rate	103.27 ± 11.71	104.93 ± 13.66	-0.52	0.61	-8.11 ~ 4.79
LogRMSSD	3.03 ± 0.57	2.66 ± 0.67	2.34	< 0.05	0.05 ~ 0.68
LogSDNN	3.34 ± 0.42	2.97 ± 0.48	3.26	< 0.01	0.14 ~ 0.60
LogLF/HF	0.78 ± 0.61	1.17 ± 0.79	-2.14	< 0.05	-0.74 ~ -0.02

### 3.2 Correlation analysis

Regarding the relationship between motor development and symptom presentation: Fine motor skills showed a significant positive correlation with symptom score (r = 0.46, P < 0.01), indicating worse fine motor skills were associated with more severe symptoms. Coordination was also significantly correlated with symptom score (r = 0.32, P < 0.05). The MABC-2 total score was positively correlated with symptom score (r = 0.33, P < 0.01).

Regarding the relationship between motor development and HRV: logSDNN showed a significant positive correlation with coordination (r = 0.37, P < 0.01). logRMSSD was positively correlated with coordination (r = 0.37, P < 0.01). The sympathetic/parasympathetic balance indicator logLF/HF was significantly negatively correlated with both HRV time-domain indices (with logRMSSD: r = -0.58; with logSDNN: r = -0.45; both P < 0.01), suggesting that a higher LF/HF ratio is consistent with decreased autonomic function.

Regarding the relationship between symptom severity and HRV: Symptom score was significantly positively correlated with logSDNN (r = 0.29, P < 0.05). The results of the correlation analysis are presented in **Table 2**.

**Table 2:** Correlations among ADHD symptoms, motor ability domains, and heart rate variability indicators.

Variable	1	2	3	4	5	6	7	8
1. Symptom Score	—	0.46**	0.32*	0.12	0.33**	0.18	0.29*	-0.20
2. Fine Motor	0.46**	—	0.42**	0.35**	0.79**	0.06	0.08	-0.14
3. Coordination	0.32*	0.42**	—	0.26	0.62**	0.37**	0.37**	-0.19
4. Static/Dyn. Balance	0.12	0.35**	0.26	—	0.79**	0.22	0.22	0.04
5. MABC-2 Total	0.33**	0.79**	0.62**	0.79**	—	0.24	0.23	-0.08
6. LogRMSSD	0.18	0.06	0.37**	0.22	0.24	—	0.95**	-0.58**
7. LogSDNN	0.29*	0.08	0.37**	0.22	0.23	0.95**	—	-0.45**
8. LogLF/HF	-0.20	-0.14	-0.19	0.04	-0.08	-0.58**	-0.45**	—

*P < 0.05, **P < 0.01. P-values for all correlation analyses (m=28) were corrected using the Benjamini-Hochberg False Discovery Rate (FDR) procedure. Significance markers (asterisks) indicate results that remained statistically significant after FDR correction.

### 3.3 Hierarchical regression analysis

Collinearity diagnostics indicated that all Variance Inflation Factor (VIF) values were below 5, suggesting no multicollinearity concerns. A three-step hierarchical regression analysis was performed with ADHD children’s symptom score as the dependent variable, and age, sex, fine motor skills, coordination, and the HRV index logSDNN as independent variables (**Table 3**). In Step 1, the control variables age and sex were entered into the equation; both significantly influenced the symptom score (both P < 0.05), and together explained 17.0% of the variance in symptom severity (R² = 0.17). In Step 2, fine motor skills and coordination were added; fine motor skills significantly predicted the symptom score (P < 0.05), increasing the explained variance by 12.9% (ΔR² = 0.129; ΔF = 5.190, P < 0.001), with the total explained variance rising to 29.0% (R² = 0.29). In Step 3, the HRV index logSDNN was introduced; logSDNN significantly predicted the symptom score (P < 0.01), adding a further 11.9% to the explained variance (ΔR² = 0.119; ΔF = 5.55, P < 0.001). In the final model, age, sex, motor variables, and logSDNN together explained 41.0% of the variance in ADHD symptom severity (R² = 0.41).

**Table 3:** Hierarchical regression models examining the independent contributions of motor abilities and heart rate variability to ADHD symptom severity.

Predictor	Step 1			Step 2			Step 3		
	β	t	P	β	t	P	β	t	P
Age	0.32	2.64	0.01	0.11	0.85	0.40	0.10	0.82	0.42
Sex	-0.29	-2.42	0.02	-0.26	-2.21	0.03	-0.23	-2.06	0.04
Fine Motor				0.29	2.07	0.04	0.29	2.20	0.03
Coordination				0.20	1.56	0.12	0.16	1.21	0.23
logSDNN							1.08	3.27	<0.01
F-value	5.81			5.91			6.42		
*P*-value	<0.01			<0.01			<0.01		
R²	0.17			0.29			0.41		
Adjusted R²	0.14			0.24			0.35		

## 4 Discussion

This study compared differences in motor development and autonomic nervous system function between children with ADHD and typically developing children, and further explored the predictive role of these factors on ADHD symptom severity. The main findings include: First, children with ADHD demonstrated significant deficits in fine motor skills, coordination, and overall motor development. Second, children with ADHD exhibited significantly reduced heart rate variability, manifested as lower SDNN and RMSSD, and higher LF/HF ratio, suggesting decreased parasympathetic activity and sympathetic dominance. Third, after controlling for age, sex, and motor abilities, logSDNN remained a significant independent predictor of ADHD symptom severity. In the final hierarchical regression model, age, sex, motor abilities, and logSDNN together explained 41.0% of the variance in symptom severity, with motor variables contributing an additional 12.9% and logSDNN contributing a further 11.9%.

The findings of this study show that children with ADHD scored significantly lower than the control group on fine motor skills, coordination, and the MABC-2 total score, consistent with previous research (18, 32). Existing studies note that approximately 50% of children with ADHD have clinically significant motor coordination difficulties (33, 34), indicating that motor development problems may be a comorbid feature of ADHD, not merely a consequence of inattention. The neural basis of motor difficulties may involve functional abnormalities in brain regions such as the cerebellum, basal ganglia, and prefrontal cortex, which are responsible for both motor control and executive functions (35–37). Therefore, shared neural mechanisms might underlie both motor development issues and core ADHD symptoms (38). Although the cross-sectional design limits our ability to infer causality, the results of the hierarchical regression analysis provide robust evidence for the link between motor coordination and ADHD symptoms. Specifically, after adjustment for age and sex, the addition of motor variables increased the explained variance in SWAN scores from 17.0% to 29.0% (ΔR² = 0.129), indicating that motor impairment contributed unique explanatory value beyond demographic factors. This statistical independence suggests that the neurological mechanisms underlying motor control may overlap with those governing core ADHD behaviors.

This study further corroborates alterations in autonomic nervous system function among children with ADHD. Their SDNN and RMSSD values were markedly lower than those of typically developing children, whereas LF/HF was higher, indicating reduced parasympathetic activity and heightened sympathetic activation. Such reductions in HRV reflect a diminished capacity of the autonomic nervous system to adapt to external stressors and internal regulatory demands (39, 40). This physiological pattern aligns closely with the core feature of “self-regulation difficulties” in ADHD (41). As an indicator of overall heart rate variability, SDNN is strongly associated with stress regulation and self-control capacity (42, 43). However, in the hierarchical regression model, only logSDNN emerged as a significant independent predictor of ADHD symptom severity. Therefore, the present findings support the predictive value of a specific HRV indicator rather than HRV as a whole (10). Specifically, the inclusion of logSDNN increased the model R² from 0.29 to 0.41, accounting for an additional 11.9% of the variance in symptom severity beyond age, sex, and motor performance.

Furthermore, this study found that both motor ability and autonomic function were significantly correlated with ADHD symptoms, but the role of the autonomic nervous system (particularly SDNN) was more prominent. Motor performance and HRV may reflect different yet partially overlapping neural substrates in ADHD: motor performance relies more on the cerebellum and sensorimotor systems (44), while HRV involves the prefrontal-vagal pathway (37, 45). Concurrent functional abnormalities in both systems may contribute to the complex behavioral characteristics of ADHD. Therefore, a comprehensive assessment incorporating both motor and HRV indicators could facilitate a multi-level understanding of the neurophysiological basis of ADHD.

The results of this study are consistent with previous findings: children with ADHD commonly exhibit motor coordination difficulties and lower heart rate variability. However, relatively few studies have simultaneously incorporated both types of indicators into the same predictive model. This study demonstrates that one HRV parameter, logSDNN, provided additional explanatory power for predicting ADHD symptoms, increasing the explained variance by 11.9% beyond demographic and motor variables. This result echoes the growing body of literature suggesting that autonomic nervous system function may be a core physiological mechanism underlying behavioral regulation in ADHD.

Additionally, the use of the SWAN scale, which tends to have a more normal distribution of scores, provided a more stable and reliable quantification of symptom severity, enhancing the credibility of this study’s correlation analyses and modeling results.

This study has significant clinical application value. First, greater attention should be paid to motor development issues in children with ADHD; motor assessment could be incorporated into routine ADHD screening to aid early identification and intervention. Second, HRV, particularly SDNN, shows potential as a physiological biomarker for ADHD, usable for symptom assessment and monitoring intervention effects. Existing research suggests that enhancing parasympathetic activity through HRV biofeedback, mindfulness training, aerobic exercise, etc., may positively impact behavioral improvement in ADHD (46, 47). In educational settings, understanding that children with ADHD may experience higher physiological stress and regulatory burden can help teachers provide more appropriate support.

## 5 Conclusions

This study demonstrated that children with ADHD exhibit marked deficits in motor development and autonomic nervous system regulation compared with typically developing peers. ADHD children showed significantly poorer fine motor skills, coordination, and overall MABC-2 performance, accompanied by reduced RMSSD and SDNN and an elevated LF/HF ratio, indicating diminished parasympathetic activity and heightened sympathetic dominance.

Correlation and hierarchical regression analyses further revealed that motor performance and autonomic function were associated with ADHD symptom severity; however, in the final regression model, only logSDNN emerged as a significant independent HRV predictor. The full model explained 41.0% of the variance in symptom severity, with motor variables contributing an additional 12.9% and logSDNN contributing a further 11.9% beyond age and sex. These findings highlight the central role of autonomic regulation in ADHD and indicate that motor development deficits also contribute uniquely to symptom expression.

In conclusion, the present study suggests that:

1. ADHD is characterized by concurrent impairments in motor coordination and autonomic nervous system function;
2. Among the HRV indices examined, logSDNN showed the clearest independent association with ADHD symptom severity and may serve as a promising physiological marker;
3. Integrating motor and autonomic indicators may facilitate a more comprehensive understanding of ADHD behavior.

These results provide empirical support for adopting multidimensional assessment approaches in clinical and educational settings and point toward future intervention strategies targeting motor skills and autonomic regulation.

## Data Availability

The datasets generated and analyzed during the current study are not publicly available due to the sensitive nature of the information concerning minors with a neurodevelopmental disorder and because the data are part of an ongoing longitudinal study. However, the data are available from the corresponding author upon reasonable request for academic discussion and collaboration, subject to approval from the ethics committee and data custodians. Requests to access the datasets should be directed to Yanfeng Zhang, zhangyanfeng@ciss.cn.
